# Supplementary material for: Genetic Engineering of Agrobacterium Increases Curdlan Production through Increased Expression of the crdASC Genes
Source: Microorganisms. 2023 Dec 28;12(1):55. doi: 10.3390/microorganisms12010055 (PMC10819609; doi:10.3390/microorganisms12010055)
Supplement: Supplementary file 1 [file microorganisms-12-00055-s001.zip › microorganisms-2761111-supplementary.pdf]

microorganisms

MDPI

Article

Supplementary Data for Genetic Engineering of *Agrobacterium* increases Curdlan Production through Increased Expression of the *crdASC* Genes

Matthew McIntosh

Institute of Microbiology and Molecular Biology, IFZ, Justus-Liebig-Universität, 35292, Giessen, Germany; matthew.mcintosh@mikro.bio.uni-giessen.de

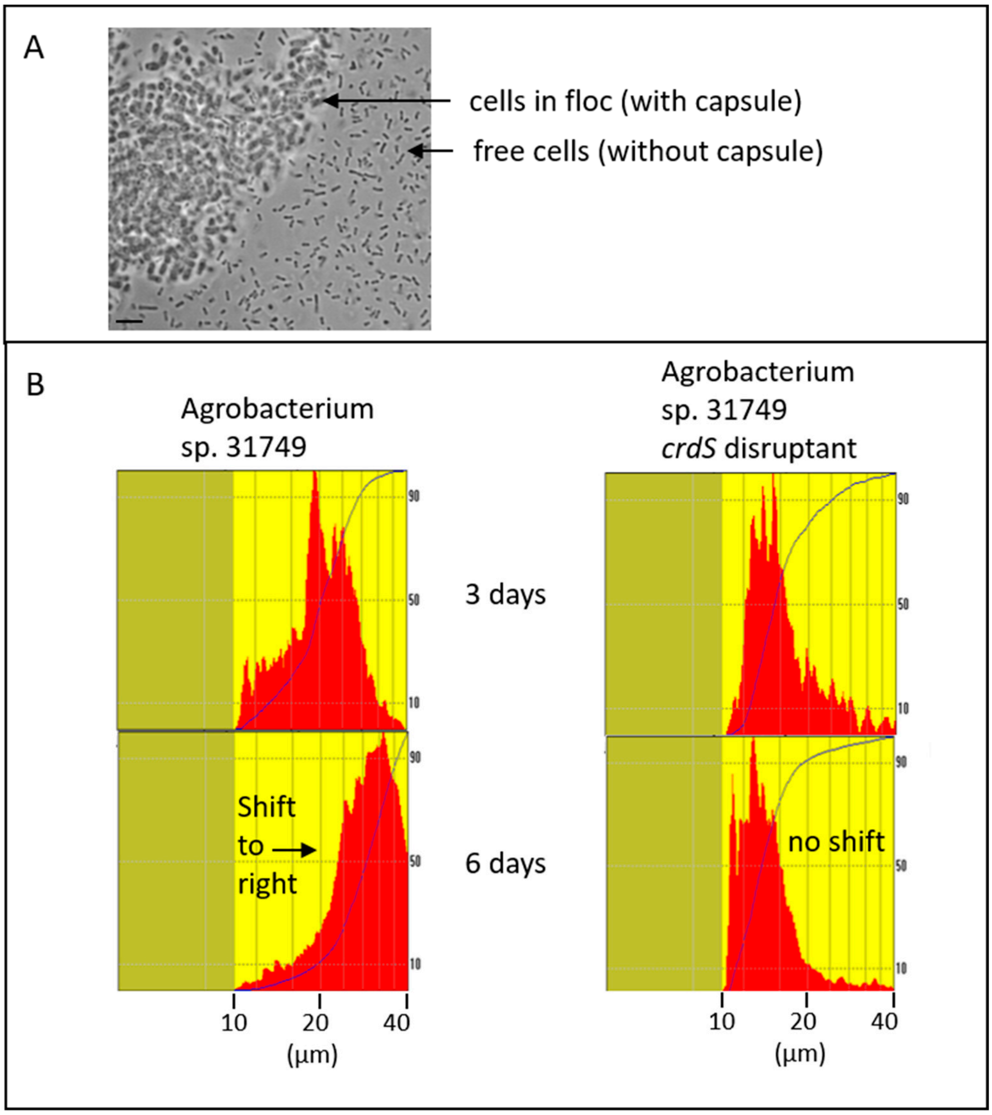

Figure S1. Curdlan promotes cell aggregation. (**A**) In liquid cultures of Agrobacterium defined broth (ADB), encapsulated cells form cell aggregates/flocs, while non-capsulated cells remain free and unattached. Bar represents 5 µm. (**B**) Liquid cultures were analysed using a particle counter with a 10-40 µm cutoff. The average size of the particles/flocs from the curdlan producing strain were clearly larger and shifted to right (compare 3 days and 6 days), while the particles from the *crdS* disruptant did not.

Supplementary File S1. The DNA sequences of the suicide plasmids used in this study.

primers and plasmids used in this study

construct name	primer name	primer sequence	application
pKPcrdA-mV	PcrdA-384H-fwd	cataagcttAGTTTTGGGAAAAAGCAACGG	suicide plasmid 1
	PcrdA+51X-rev	atgtctagaTCTTACGGATGCGCCTTTAT	(PCR for Pcrd)
pK18-crdA KO	crdA-rbs-sal-fwd	atatgtcgacaacagaggagttgatttcATGctgttcc	suicide plasmid 2
	crdA+489x-rev	tatatctagagacgagtttttgtttgcgagccg	(PCR for *crdA* fragment)
pK-PphaP-crdA	PphaP-134-H-fwd	catacaagcttGCAACGCAAGGCGTTTCGGG	suicide plasmid 3
	PphaP-ts-Sal-rev	catagtcgacTCTCCTGCGAGCGGGCTCAT	(PCR for PphaP)
	crdA-rbs-sal-fwd	atatgtcgacaacagaggagttgatttcATGctgttcc	
	crdA+489x-rev	tatatctagagacgagtttttgtttgcgagccg	(PCR for *crdA* fragment)
pPHU-Pcrd::mVenus	PcrdA-384H-fwd	cataagcttAGTTTTGGGAAAAAGCAACGG	reporter plasmid
	PcrdA+51X-rev	atgtctagaTCTTACGGATGCGCCTTTAT	(PCR for Pcrd)
pPHU-PphaP::mVenus	P0381-H-fwd2	gataaagcttAATACGGCCATTACACCAAC	reporter plasmid
	P0381-X-rev2	catatctagaGTCCTGCATCACTTTGCTG	PCR for PphaP, 0381 = *phaP*)
	mVenus-E-rev	catgaattcTTACTTGTACAGCTCGTCCATGC	reporter, mVenus gene
	mVenus-X-fwd	tcatctagaATGGTGAGCAAGGGCGAGGAGCT	(PCR for mVenus)
pK18-crdS KO	CRDS1.2	CGGGATCCTCTAGACGACTTCATCATGGTCCTC	suicide plasmid
	CRDS5.2.2	CGGGATCCTCTAGACCATCAGGCGATATGTCAG	(PCR for *crdS* fragment)

DNA sequences relevant for suicide and reporter plasmids

Colour coded key:
Vector sequence (pK18mob2)
crdA fragment
PcrdA fragment
mVenus sequence
PphaP
atg =start codon of *crdA*
various restriction digest sites

> pK18PcrdA-mV, Suicide plasmid 1 (reporter plasmid for 31749)

ACGACGGCCAGTGCCAAGCTTAGTTTTGGGAAAAAGCAACGGCACTTCCATACCGACTT
GAGGCGCTAACGCGCTTGCGGTCTGGAACTTGACCTACCCGGCGGTAGGTCTTTGCGACG
GCATGCCGTCGCCCCCATAAGTTGCATATGCGAAATGGCGAAGCCAGGGTGTGTTTTCCA
AGCACGCCATTTAAAAGGCAATTCTAATATGAAACGATTGAAATTTCGTCTTTCAAATTC
TCTTCGATGAATTTTCGCGTCCCCAAAATCGTCAGATGCCTATTTGTTGGTCAAAATTGGA
AAATTAGTTAATGCAATTTTTACTATGTTGCGGCGCACAAGAACTTCATTCACCATTTCAA
TACTGCGGGAGGAACGGTTTCTCAACAACAGAGGAGTTGATTTCatgCTGTTCCGCAATAA
GCCTGACGCAAAATATAAAGGCGCATCCGTAAGATCTAGAATGGTGAGCAAGGGCGAG
GAGCTGTTCACCGGGGTGGTGCCCATCCTGGTCGAGCTGGACGGCGACGTAAACGGCCA
CAAGTTCAGCGTGTCCGGCGAGGGCGAGGGCGATGCCACCTACGGCAAGCTGACCCTGA
AGCTGATCTGCACCACCGGCAAGCTGCCCGTGCCCTGGCCCACCCTCGTGACCACCCTGG
GCTACGGCCTGCAGTGCTTCGCCCGCTACCCCGACCACATGAAGCAGCACGACTTCTTCA
AGTCCGCCATGCCCGAAGGCTACGTCCAGGAGCGCACCATCTTCTTCAAGGACGACGGC
AACTACAAGACCCGCGCCGAGGTGAAGTTCGAGGGCGACACCCTGGTGAACCGCATCG
AGCTGAAGGGCATCGACTTCAAGGAGGACGGCAACATCCTGGGGCACAAGCTGGAGTA
CAACTACAACAGCCACAACGTCTATATCACCGCCGACAAGCAGAAGAACGGCATCAAG
GCCAACTTCAAGATCCGCCACAACATCGAGGACGGCGGCGTGCAGCTCGCCGACCACTA
CCAGCAGAACACCCCCATCGGCGACGGCCCCGTGCTGCTGCCCGACAACCACTACCTGA
GCTACCAGTCCAAACTGAGCAAAGACCCCAACGAGAAGCGCGATCACATGGTCCTGCTG
GAGTTCGTGACCGCCGCCGGGATCACTCTCGGCATGGACGAGCTGTACAAGTAAGAATT
CGTA

> pK18PcrdA-mV, Suicide plasmid 1 (reporter plasmid for C58)

ACGACGGCCAGTGCCAAGCTTAGTTTTGGGAAAAAGCAACGGCACTTCCATACCGACTT
GAGGCGCTAACGCGCTTGCAGTCTGGAAATTGACCTACCCGGCGGCAGGCCTTTGCGAC
GGCATGCCGTCGCCCCCATAAGTTGCATATGCGAAATGGCGAAGCCAGGGTGTGTTTTCC
AAGCACGCCATTTAAAAGGCAATTCTAATATGAAACGATTGAAATTTCGTCTTTCAAATT
CTCTTCGATGAATTTTCGCGTCCCCAAAATCGTCAGATGCCTATTTGTTGGTCAAAATTGG
AAAATTAGTTAATGCAATTTTTACTATGTTGCGGCGCACAAGAACTTCATTCACCATTTCA
ATaCTGCGGGAGGAACGGTTTCTCAACAACAGAGGAGTTGATTTCatgCTGTTCCGCAATA
AGCCTGACGCAAAATATAAAGGCGCATCCGTAAGATCTAGAATGGTGAGCAAGGGCGA
GGAGCTGTTCACCGGGGTGGTGCCCATCCTGGTCGAGCTGGACGGCGACGTAAACGGCC
ACAAGTTCAGCGTGTCCGGCGAGGGCGAGGGCGATGCCACCTACGGCAAGCTGACCCTG
AAGCTGATCTGCACCACCGGCAAGCTGCCCGTGCCCTGGCCCACCCTCGTGACCACCCTG
GGCTACGGCCTGCAGTGCTTCGCCCGCTACCCCGACCACATGAAGCAGCACGACTTCTTC
AAGTCCGCCATGCCCGAAGGCTACGTCCAGGAGCGCACCATCTTCTTCAAGGACGACGG
CAACTACAAGACCCGCGCCGAGGTGAAGTTCGAGGGCGACACCCTGGTGAACCGCATCG
AGCTGAAGGGCATCGACTTCAAGGAGGACGGCAACATCCTGGGGCACAAGCTGGAGTA
CAACTACAACAGCCACAACGTCTATATCACCGCCGACAAGCAGAAGAACGGCATCAAG
GCCAACTTCAAGATCCGCCACAACATCGAGGACGGCGGCGTGCAGCTCGCCGACCACTA
CCAGCAGAACACCCCCATCGGCGACGGCCCCGTGCTGCTGCCCGACAACCACTACCTGA
GCTACCAGTCCAAACTGAGCAAAGACCCCAACGAGAAGCGCGATCACATGGTCCTGCTG

GAGTTCGTGACCGCCGCCGGGATCACTCTCGGCATGGACGAGCTGTACAAGTAAGAATT
CGTA

>pK18-crdA KO, Suicide plasmid 2 (KO plasmid for C58)
ACGACGGCCAGTGCCAAGCTTGCATGCCTGCAGGTCGACAACAGAGGAGTTGATTTCatg
CTGTTCCGCAATAAGCCTGACGCAAAATATAAAGGCGCATCCGTAAGATTAGCCTCCTAT
ACTGTTCTCGGTGTGGTGGCGACGGTGTTTTCTTCTTCCGTGTTTCCGCCTGTATTCACCTC
TCAAACGAGCCGTGCTCTGATCAATGCCCCCATTATTCCCTTGACGACGCCGATCAGCGG
CGTTGTGAAAACATCGAACGACAGGGGCGGTGCCACCATCGAGAACGACAAGGTGGAC
AATACAACGCTGATCGGCCTGAAGGTTCAACTGGCGGCGCTTGACAATGAGATGCGCCA
GAAGAACTCGATCGTTTCGGACTATGCCTCGCGGATCGACGACCTGAACAGGGATCTTTC
CAGCCAGCAGGCGGCTTTGCTGCTGCGGACAGAGTCCGATCTCGAGGCTGCAGAGGCGG
CCTTGCAGATGGTTACCTATTCCACGCGGATCGCAAAAGCCGAAGCGGCTCGCAAACAA
AAACTCGTCTCTAGAGGATCCCCGGGTACCGAGCTCGAATTCGTA

>pK18-PphaP-crdA, Suicide plasmid 3 (over-expression plasmid for 31749)

AAGCTTGCAACGCAAGGCGTTTCGGGCCAGGTGCGAAAAAATGCTGCGGTGCAGAATGA
GCGGTTGATTTTCTGCGGCGCAGCATTTATATCCTTGTCACAGGATAACCGGGCATGAGC
CCGCTCGCAGGAGAGTCGACAACAGAGGAGTTGATTTCatgCTGTTCCGCAATAAGCCTG
ACGCAAAATATAAAGGCGCATCCGTAAGATTAGCCTCCTATACTGTTCTCGGTGTGGTGG
CGACGGTGTTTTCTTCTTCCGTGTTTCCGCCTGTATTCACCTCTCAAACGAGCCGTGCTCTG
ATCAATGCCCCCATTATTCCCTTGACGACGCCGATCAGCGGCGTTGTGAAAACATCGAAC
GACAGGGGCGGTGCCACCATCGAGAACGACAAGGTGGACAATACAACGCTGATCGGCC
TGAAGGTTCAACTGGCGGCGCTTGACAATGAGATGCGCCAGAAGAACTCGATCGTTTCG
GACTATGCCTCGCGGATCGACGACCTGAACAGGGATCTTTCCAGCCAGCAGGCGGCTTT
GCTGCTGCGGACAGAGTCCGATCTCGAGGCTGCAGAGGCGGCCTTGCAGATGGTTACCT
ATTCCACGCGGATCGCAAAAGCCGAAGCGGCTCGCAAACAAAAACTCGTCTCTAGAGG
ATCCCCGGGTACCGAGCTCGAATTCGTA

Disclaimer/Publisher's Note: The statements, opinions and data contained in all publications are solely those of the individual author(s) and contributor(s) and not of MDPI and/or the editor(s). MDPI and/or the editor(s) disclaim responsibility for any injury to people or property resulting from any ideas, methods, instructions or products referred to in the content.
